# Child Training as an Exact Science

**Published:** 1916-12

**Authors:** Malone Duggan


### Book Reviews.

CHILD TRAINING AS AN EXACT SCIENCE. By George W. Jacoby, M. D. Published by Funk & Wagnalls Co. 1914.

"The child is father to the man" is an often quoted aphorism, and emphasizes the important rôle that the child plays in our social progress. Never before, in the history of civilization, has child training played so conspicuous a part in our educational system. Much of this is due to the better understanding of psychology and its application to the growth of the child. Any pedagogic method that fails to utilize psychologic laws fails in its purpose. But the distinction between pathological and normal psychology is not always differentiated, though it is of the most essential importance. It is this distinction that Dr. Jacoby seeks to emphasize. He takes the view that every psychic manifestation of life must be accompanied by a physical movement, or a change in the central nervous system. The pedagogue and physician should combine their efforts in harmonious co-operation to the end that this differentiation of physiological and pathological psychology is made the common ground from which to develop the child.

Besides giving a historical survey of the subject the author proceeds with giving a résumé of the nervous system and its functions developing the normal psychology. Then he takes up the abnormalities, functional and organic, and concludes with admirable suggestions on the prophylactic and therapeutic training.

Particular emphasis is placed on the management of educatable and uneducatable. It is such a book as the physician needs to give him suggestions on how he may meet the growing demand for this sort of advice that his position in society imposes.

MALONE DUGGAN.

